# Exploring relational and emotional experiences of the LGBTQ+ community through a cognitive analytic therapy lens

**DOI:** 10.1111/papt.70016

**Published:** 2025-11-29

**Authors:** Deborah Charis Bell, Rowan Tinlin‐Dixon

**Affiliations:** ^1^ Newcastle University Newcastle upon Tyne UK; ^2^ Cumbria, Northumberland, Tyne and Wear NHS Foundation Trust Newcastle upon Tyne UK

**Keywords:** affirmative therapy, cognitive analytic therapy, LGBTQ+, mental health, minority stress, relationships, sexuality

## Abstract

**Background:**

Cognitive analytic therapy (CAT) offers a relational framework for understanding psychological difficulties, emphasising how early relational and socio‐cultural experiences are internalised and shape the self through a repertoire of reciprocal roles (RRs).

**Aims:**

This qualitative study seeks to explore the relational experiences of LGBTQ+ individuals using CAT theory, focusing on identity development (self–self relationships), self–other relationships, and self–society relationships.

**Methods:**

Interpretative phenomenological analysis (IPA) guided interviews with 11 participants. CAT theory was applied as an additional framework to provide a clinically applicable understanding of these themes.

**Results:**

Six group experiential themes (GETs) were identified: *Coming Out*: *Discovering and Sharing Who I Am*; *Is it Wrong to be LGBTQ +? Questions and Beliefs about Being ‘Normal’*; *Different Parts of Me*; *Feeling Safe in Relation to Others*; *Feeling Unworthy and Unlovable*; and *Being Authentically Me*. Illustrations of themes were created. A CAT map was developed from the themes, outlining RRs, reciprocal role procedures (RRPs), and self‐states. The findings highlight the influence of early caregiving relationships, the coming out process, and societal experiences on participants' self‐development.

**Conclusion:**

The CAT map captures the distinct RRs and procedures that the LGBTQ+ community may experience, emphasising the critical role of the self–society relationship in identity formation. It underscores the importance of the ‘healthy island’ concept and illustrates how healing RRPs can promote a more integrated and connected sense of self. This framework may help clinicians support LGBTQ+ individuals facing mental health challenges related to their relational experiences and sense of self.

## INTRODUCTION

### The construction of sexual identity

Language is often subjective; however, from an academic perspective, Gordon and Silva (2015) define sexual orientation as the array of sexual attractions made up of thoughts, feelings, and behaviours, and sexual identity as how we make sense of this array of experiences. Sexual identity is integrated into identity formation and the view of the self (Gordon & Silva, 2015), and is the focus of the current study.

As Queer Theory evolved, it challenged binary frameworks of sexual identity formation, emphasising the importance of societal constructs in shaping identity. Rust (1993) explored sexual identity formation from a social constructionist perspective, highlighting the influence of societal and cultural factors in shaping experiences. Gordon and Silva (2015) build on this concept and proposed that sexual identity is dynamic and multifaceted; meaning it is shaped by an interplay of individual experiences, social and cultural narratives, and interpretive processes. One such interpretive process is intersectionality. Race, class, gender, age, and other nonsexual identities can influence how we interpret internal processes, which in turn influences how identity is expressed (Gordon & Silva, 2015). This highlights the potential for identity conflicts to arise due to non‐affirming contexts, which can lead to sexuality being viewed as incompatible with other identities (Gordon & Silva, 2015).

Another interpretative process is others' response to one's sexual identity. Stryker and Burke (2000) emphasised social support in identity formation, noting that when an aspect of identity is affirmed, it becomes more significant. This highlights the connections between sexual identity development, coming out, and acceptance. Furthermore, acceptance from significant others has been found to offset the effects of discrimination (Coleman, 1982).

It is evident that sexual identity development and coming out are a complex process, influenced by intersectionality, relational feedback, and environmental factors pertaining to safety and connecting with one's whole self.

### The cognitive analytic therapy lens

Cognitive analytic therapy (CAT) is an object relations‐based approach to cognitive therapy that emphasises the internalisation of early relational experiences (Ryle & Kerr, 2020). Drawing on Vygotskian and Bakhtinian concepts, CAT has a focus on the social formation of the mind, highlighting how meaning, identity, and psychological distress are shaped through dialogue and relationships with others (Ryle & Kerr, 2020).

CAT emphasises how early relational experiences are internalised, creating The Self through an internalised repertoire of reciprocal roles (RRs) (Ryle & Kerr, 2020). CAT views the Self as a ‘dynamic fragment of a social whole’, emphasising the multiple parts influenced by relational, social and cultural factors (Ryle & Kerr, 2020, p. 17). This development is shaped through language and the process of meaning‐making, which mediates how individuals understand themselves and their relationships (Ryle & Kerr, 2020). This aligns with Gordon and Silva's (2015) interpretive theory of sexual identity development, as both emphasise the importance of meaning‐making derived from experiences and in dialogue with others.

CAT provides a relational understanding of strengths and difficulties in the form of helpful and unhelpful relational patterns and procedures. CAT understands how coping patterns, or reciprocal role procedures (RRPs), emerge as the Self learns behaviours to respond to relational experiences (Ryle & Kerr, 2020). If a person has a traumatic relational experience, it can result in problematic RRPs which impact relationships with the Self, others, and society. Therefore, CAT's emphasis is on recognising problematic RRPs and enabling change.

CAT theory has recently been used to help understand sexuality and draw attention to the harmful RRPs experienced by some LGBTQ+ individuals, which can become internalised, leading to a ‘disconnected self’ (Tinlin‐Dixon, 2023). There is an opposition between the disconnected and authentic Self‐states, and the CAT framework illustrates how experiencing affirming RRs can create a safe space for the authentic Self to be integrated (Tinlin‐Dixon, 2023).

As Benson and Discepolo Ahmadi (2024) note, it is important to situate the Self, particularly aspects such as sexual identity, within broader political contexts. The CAT framework supports this by recognising the influence of political and societal norms, alongside biological and psychological factors, which can be meaningfully explored through tools like the CAT map and reformulation letter (Benson & Discepolo Ahmadi, 2024). Therefore, the CAT model seems suitable to explore relational experiences of the LGBTQ+ community, as sexuality has historically been situated and defined in a socio‐cultural and political context. Previous literature has emphasised the significance of intersecting influences of identity, such as social and cultural contexts and the individuals' own meaning‐making (Gordon & Silva, 2015; Rust, 1993). Therefore, the CAT model provides an appropriate framework to further expand on how these experiences have impacted self–self and self–other relationships.

### Aims and objectives

This primary qualitative research aims to explore the relational experiences of LGBTQ+ individuals, including early experiences, development of the Self, and sharing sexual identity with others.

1. Understand how early caregiving relationships impact an individual's relationship with the Self and others.
2. Understand how relationships are impacted through the coming out process, or how coming out is impacted by relationships.
3. Understand how other aspects of identity relate to sexual identity and our relationships.
4. Understand how sexual identity and relational experiences impact the Self.

## METHODOLOGY

### Approach

#### Ontology

This research adopts a relativist ontological position, recognising that knowledge, truth, and morality are shaped by experiences within social and cultural contexts (Hume, 2000). Favouring contextualism, this research recognises that knowledge is influenced by perspective, making it dynamic and context‐dependent (Larkin et al., 2006; McKenna, 2015). This is pertinent to cultural shifts in societal perspectives of LGBTQ+ relationships.

#### Epistemology

This research takes a social constructionist epistemological approach, emphasising that knowledge is actively constructed through social processes and cultural contexts (Burr, 2004; Gergen, 2001). This aligns with Ryle and Kerr's (2020) concepts in CAT regarding the social formation of the mind, which emphasises social and relational influences in the development of self.

### Interpretative phenomenological analysis (IPA)

IPA is a qualitative methodology committed to how people make sense of their experiences, and is concerned with psychological questions and lived experience (Smith et al., 2022).

### Public involvement

A focus group (*n* = 3) and survey (*n* = 8) with LGBTQ+ individuals was conducted to discuss interview questions, research aims, and language use. The group identified that LGBTQ+ was the preferred and most inclusive term for referring to the community. The focus group gave feedback on the interview schedule and made suggestions about the order, which was taken on board. This demonstrates how the hermeneutic circle guided interview development, as input from the LGBTQ+ community ensured each question (the part) captured meaningful experiences and was aligned with the research question (the whole).

### Recruitment and participants

#### Ethical considerations

Ethical approval was granted by Newcastle University's Research Ethics Board (REF: 2587/33513). Confidentiality, consent, and the purpose of the research were discussed with participants prior to interview, and debriefs were provided.

#### Recruitment

Recruitment was opportunistic, and a purposive sampling framework ensured diversity in age, ethnicity, gender, religion, socio‐economic status, level of education, employment, geography, and sexual identity within the sample. This strategy, ensured marginalised perspectives within the LGBTQ+ community, could be intentionally included to increase insight (Levitt et al., 2017).

#### Participants

Sample size was determined by the model of information power (Malterud et al., 2016). All participants (*n* = 11) were over 18 years old, had the capacity to consent, and identified as LGBTQ+. Participants were required to speak English (but not necessarily as a first language) as analysing semantic content requires an in‐depth knowledge of the linguistic context.

Table 1 (given below) shows the socio‐demographic characteristics of participants (pseudonyms given). Level of education, geography, socio‐economic status, and employment demographics are not reported here to protect anonymity.

**TABLE 1:** Characteristics of participants.

Name	Age	Gender	Ethnicity	Sexuality	Religion
Alex	25–29	Non‐Binary Trans‐Masculine	White British	Gay	Not sure
Alistair	25–29	Male	Black African	Gay	Christian
Aysha	35–39	Female	White British	Gay	Christian
Asher	25–29	Non‐Binary	Asian Pakistani	Gay	Christian
Ben	30–34	Male	White British	Pansexual/demisexual	Atheist
Grace	50–54	Female	White British	Unsure	Christian
Jenny	35–39	Cis‐Woman	White British	Lesbian	No religion
Liam	25–29	Non‐Binary Trans‐Masculine	White British	Queer	No religion
Oliver	30–34	Male	White British and Welsh	Bisexual	Not sure
Theo	25–29	Non‐Binary	Any Other White Background	Gay/queer	Not sure
Tracy	30–34	Female	Black British	Bisexual	Christian

### Procedure

A semi‐structured interview process was used, divided into three parts: Coming Out, Early Relationships, and Relationships with Others, and Relationship with Self and Sense of Identity. Interviews took place online via Microsoft Teams.

### Analysis

#### IPA

Data was analysed using IPA, outlined by Smith et al. (2022). Interviews were transcribed and coded and grouped into patterns of meaning, using NVivo. To increase the accessibility of the findings, an artist created images which are a visual interpretation of each group experiential theme (GETs).

#### Participant feedback

Considering the hermeneutic circle and the dialogical influence of CAT, the findings (images and themes) were explored with participants after the study was complete. This allowed participants to recognise their own voice and see how it related (or differed) to others, highlighting the research's idiographic and phenomenological focus.

Integration of CAT theory was used to deepen the understanding of the GETs constructed and provide a clinically applicable framework; this was embedded in three ways. First, a map was co‐created with CAT clinicians and researchers (*n* = 3) using the GETs, identifying RRs and self‐states evident in the data. In CAT, the map is a collaboratively created therapeutic tool that visually organises key RRs and RRPs (Potter, 2020). It serves as an anchor for the therapeutic process, offering a shared framework that creates space for reflection, observation, and understanding of relational patterns (Potter, 2024). Here, the map also acted as the ‘whole’ within the hermeneutic circle, and each participant may resonate with parts of the map, meaning that the individual voice will not be lost.

Second, in CAT, letters help summarise and share meaning within the reformulation stage. Reformulation is a process in which a new understanding of difficulties or experiences is found, and highlights ways in which individuals learned to survive and get their needs met as a result of some of their early life experience (Jenaway, 2024). A group reformulation letter was written to participants, capturing the collective target problems, RRs, and RRPs.

Third, endings are a significant aspect of CAT, providing an opportunity to enact a new role of ‘ending well’ (Ryle & Kerr, 2020); therefore, reviewing GETs in a 1–1 session with the researcher was offered to mark the end of the research.

### Methodological rigour

This research was guided by Yardley's (2000) principles for assessing the quality of qualitative methodology in psychological research, which are sensitivity to context, commitment and rigour, transparency and coherence, and impact and importance.

To maintain sensitivity to context, the authors explored LGBTQ+ history and popular culture and reflected on the power dynamic inherent to interviewing. They utilised Bourdeau's (2000) principles to minimise harm through the use of bracketing interviews, reflexivity journals, providing clarity on the role of the interviewer versus clinician and research team debriefs.

The researchers embedded methodological rigour by becoming immersed in the methodology and data, which reflects the idiographic nature of IPA. The data analysis was explored in triangulation with research supervisors, CAT practitioners, members of the LGBTQ+ community, and participants, which ensured commitment to true construction of meaning.

Transparency was increased through NVivo as a digital audit trail was kept, enhancing the confidence in findings. Additionally, GETs are supported by quotes and a visual interpretation helps convey the complexity of the phenomenon (Levitt et al., 2017). A reflexive journal and reflexivity statement were used to acknowledge the researcher's own context.

Finally, the research aimed to achieve clinical impact to help clinicians better support the LGBTQ+ community in therapeutic spaces by gaining a deeper understanding of the unique and shared experiences of LGBTQ+ individuals.

### Researcher reflexivity statement

The primary author (DB) identifies as White, female, and straight, and acknowledges that her personal background shapes perspectives and lived experience. She reflects that her identity holds privileges, which distance her from participants' experiences.

The second author (RTD) identifies as a White queer *cis*‐woman, working clinically with gender‐diverse people and researching LGBTQ+ mental health. As a member of the LGBTQ+ community and an affirmative clinician, practising reflexivity was key to monitoring the relationship between the personal and the professional.

Throughout the research process, they have been mindful of positionality and potential biases. It has been a delicate oscillation of feeling separate and feeling similar as they connected to different relational interactions. CAT‐informed research supervision supported noticing, observation, and mapping the research process.

## RESULTS

### Group experiential themes

Participants made sense of their relationships through dialogue with the researcher. Six GETs and subthemes, detailed in Table 2 (shown below), were identified. Visual interpretations of each theme were created and are shown in the relevant subsection (see Figures 1, 2, 3, 4, 5, 6, 7, 8, 9, 10, 11, 12, 13, 14, 15 below) and collectively as a storyboard shared more widely.

**TABLE 2:** GETs and subthemes.

GET	Subtheme
Feeling Threatened	Trying to survive and ways to escape
Coming Out: Discovering and Sharing Who I Am	A tiring process of finding a label The pain of being rejected and feeling alone
Is it Wrong to be LGBTQ+? Questions and Beliefs about being ‘Normal’	Doubting myself Wishing I was straight
Different Parts of Me	Rejection from church versus reconciliation with God
Feeling Unworthy and Unlovable	
Being Authentically Me	Feeling free versus fitting in Relearning relationships through experiencing safety and acceptance Finding strength in myself versus finding strength in community

#### Feeling threatened

This GET exemplifies participants' feeling of safety within relationships due to their sexual identity, including early caregiver and romantic relationships. Participants recounted feelings of loneliness and fear during their childhood, which occurred as a result of discrimination and rejection due to their sexuality. Ben described hypervigilance, stating, ‘I'm so afraid that I'm going to get punched in the face again.’

Other participants did not recall abuse but shared feeling afraid and lonely due to lack of support as support systems became strained due to family and friends not approving of their sexuality. These experiences of feeling unsupported, uncared for, and misunderstood highlight why many participants felt lonely and afraid growing up.

**FIGURE 1:**
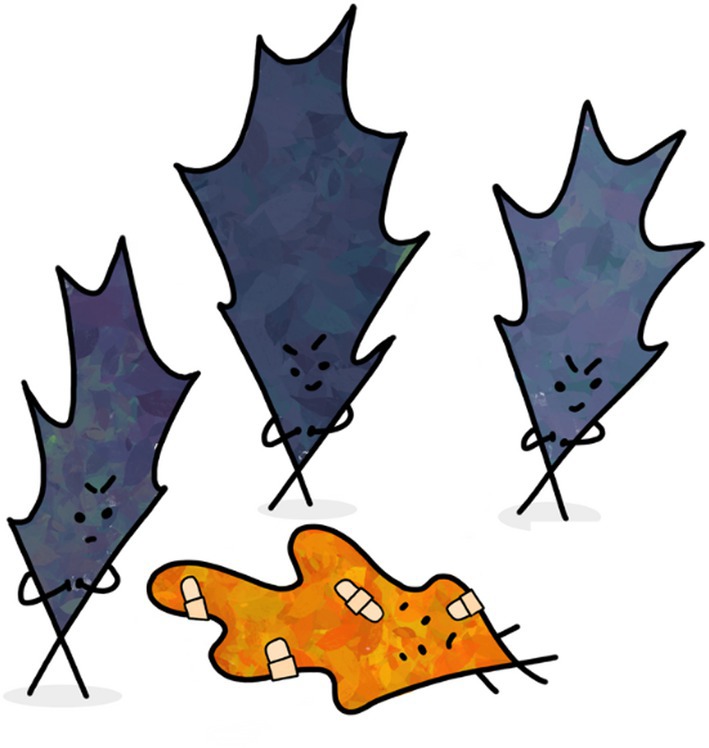
Feeling threatened.

##### Trying to survive and ways to escape

In response to the subtheme of ‘Feeling Threatened’, participants developed coping strategies to manage distress related to their sexuality.

For some, self‐harm provided an emotional release in the absence of supportive and affirming relationships. Social withdrawal was also a coping strategy. Asher found refuge in social media and Netflix, as avoiding social interactions reduced the risk of rejection from others. Similarly, Alistair, Tracy, and Jenny withdrew from unsafe friendships where their sexuality was not affirmed, highlighting that prioritising safety was necessary and confronting their friends felt overwhelming.

Numbing was another survival strategy. Theo said that substances helped him ‘drown my sorrows’ and alleviate feelings of shame associated with his sexuality. Jenny used substances to navigate social situations, masking her autism and sexuality to fit into heteronormative and neurotypical environments. She explained that substances helped her manage feelings of depression, frustration, and boredom, thus facilitating social interactions.

Overall, these mechanisms highlight the survival strategies participants employed to manage their painful experiences.

**FIGURE 2:**
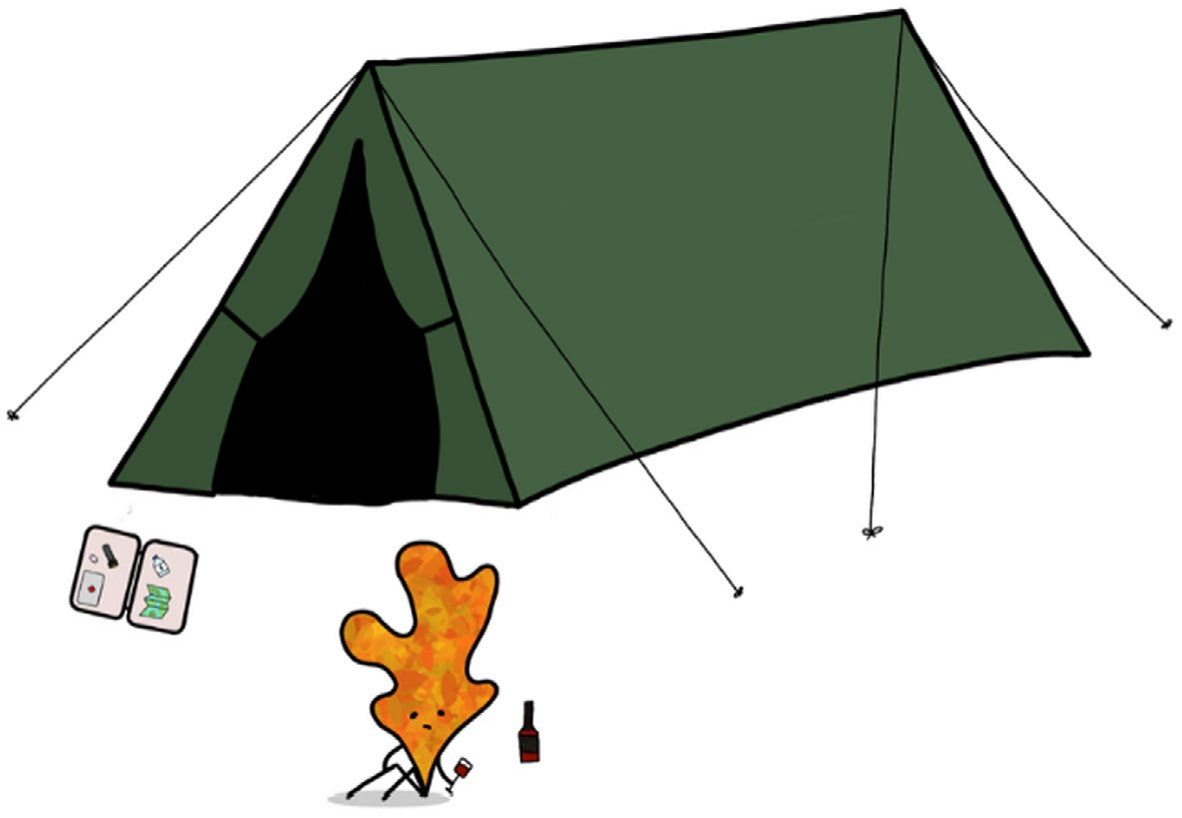
Trying to survive and ways to escape.

#### Coming out: Discovering and sharing who I am

This GET captures the varied experiences of disclosing sexual identity.

##### A tiring process of finding a label

For some participants, coming out is a multi‐staged, non‐linear process, described as ‘coming in and out of the closet.’ The closet represents both safety and inauthenticity, creating a tension between the desire to be open and the need for security.

Participants described challenges finding a label, likening the process to ‘trying on hats’ as they navigated stages to understand their identity, in an attempt to prioritise connecting with others. Ben and Theo initially identified as bisexual, finding it more socially acceptable and ‘easier to come back from’, highlighting societal pressure to conform to heteronormativity.

Aysha describes her experience by saying, ‘'I keep coming out every time’, highlighting it as a repetitive process. Aysha and Jenny emphasise the exhaustion of continually assessing the safety of environments and relationships while navigating the conflict between authenticity and security.

**FIGURE 3:**
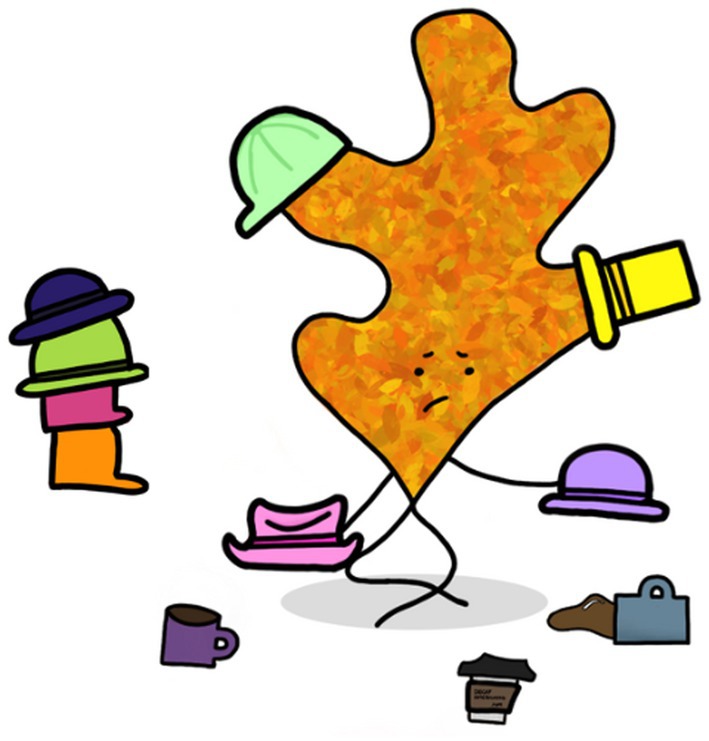
The tiring process of finding a label.

##### The pain of being rejected and feeling alone

Seven participants described feeling rejected by friends and family. Some felt unheard or unaccepted, leading to feeling unloved and unable to be authentic. For example, Theo shared how his sexuality was dismissed (‘You think you're gay, but you're not really gay’), leaving him feeling invalidated and insignificant.

Family reactions were significant as these tended to be the people participants came out to first. Ben anticipated rejection, preparing to be ‘disowned’ and feared an angry response as if his sexuality would be punished. Grace feared being a disappointment to her parents, expressing a desire for their approval and described her parents' reaction as ‘they tolerated it’, suggesting reluctant acceptance.

Aysha speaks about an ongoing rejection from the church community and says:

People, you know, they recoil that you're talking about your wife, they recoil and you're just like, 'oh, that's quite painful.

For some participants, these reactions meant participants made decisions to end relationships, which involved a grieving process. Visceral terms like ‘cut off’ and ‘broke’, conveyed feelings of loss and hurt.

These experiences illustrate the rejection participants experienced as others treated their sexuality as unpleasant, causing feelings of fear, disappointment, and loneliness.

**FIGURE 4:**
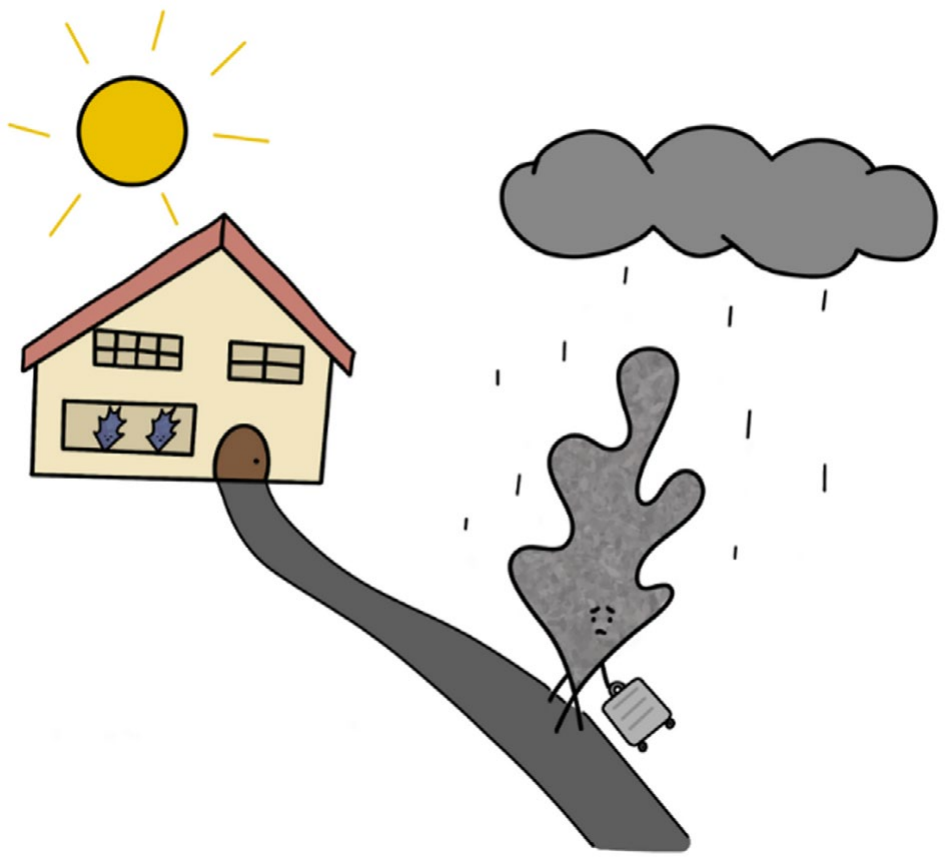
The pain of being rejected and feeling alone.

#### Is it wrong to be LGBTQ+? questions and beliefs about being ‘normal’

##### Doubting myself

Several participants stated that societal reactions led to self‐doubt. Asher reconsidered his decision to come out, contemplating a ‘return’ to a ‘normal [straight]’ life. Similarly, Tracy thought she should ‘retrace her steps.’ This notion of ‘going back’ implies they questioned their sexual identity, contemplating conforming to heterosexual norms to appease others. Asher's reference to ‘normal’ emphasises the influence of societal views that regard heterosexuality as the default, highlighting the impact of internalised homophobia.

Grace's uncertainty about whether she had ‘done the right thing’ by disclosing her love for a woman to her parents highlights the significance of parental reactions. For Asher, Grace, and Tracy, their parents' reactions impacted their view of themselves, suggesting that affirmation from parents plays an important role in their acceptance of sexuality.

Aysha's and Jenny's self‐doubt arose from feelings of isolation and questioning their differences due to homophobia. This resonates with Asher's perception of heterosexuality as the norm, reflecting a heteronormative environment that heightened their sense of difference.

##### Wishing I was straight

This subtheme highlights the impact of internalised homophobia, as participants shared a desire to be heterosexual. Oliver described suppressing his sexuality and altering his behaviours to conform to a heterosexual appearance, stating, ‘I don't want to be that.’ The use of the demonstrative pronoun and the impersonal reference implies rejection of his sexuality because being gay is undesirable.

Similarly, Theo felt compelled to ‘go back in the closet’ after a therapist suggested he change his voice. Both Theo and Ben felt guilty about their sexuality and expressed a wish to be straight in their youth. Their narratives reveal a perception of their sexuality as a burden, with Ben calling a hypothetical straight identity ‘delightful’, an adjective that suggests a longing for the happiness and ease associated with being heterosexual.

Their experiences highlight the impact of society's heteronormative culture, which privileges heterosexuality and results in fewer societal discriminations for those who conform.

**FIGURE 5:**
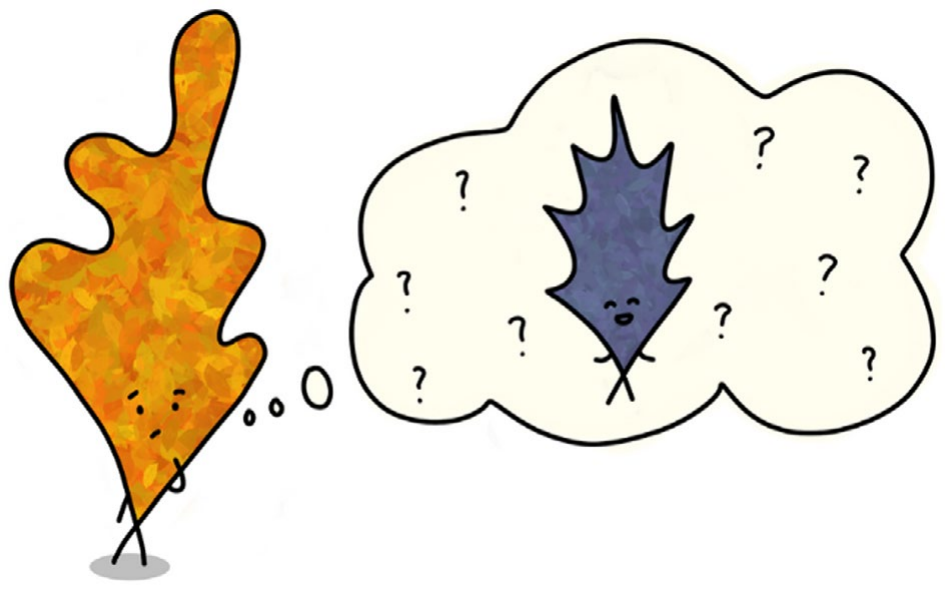
Doubting myself and wishing I was straight.

#### Different parts of me

Whilst sexuality was an integral part of each participant's sense of self, there were other identity aspects that were important; however, at times these felt incompatible, particularly religion.

**FIGURE 6:**
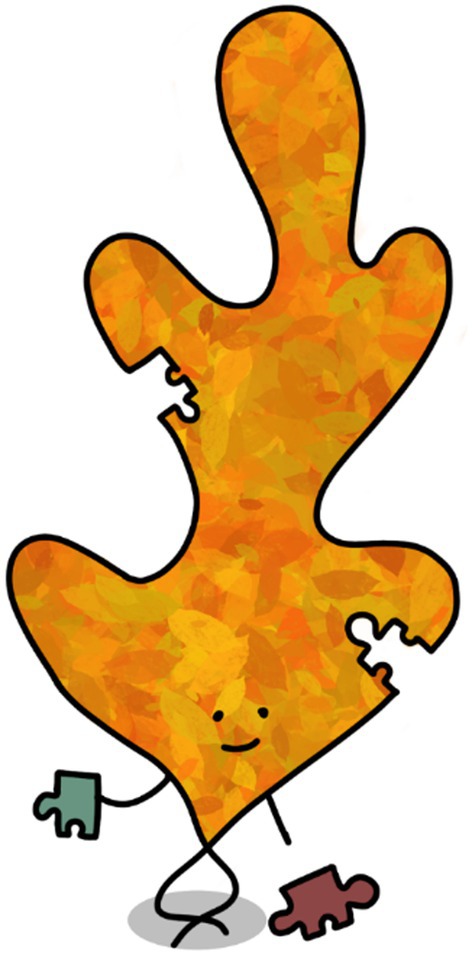
Different parts of me.

##### Rejection from church versus reconciliation with God

For five participants, faith was fundamental to their identity. Their shared experiences created a narrative of distress and ambiguity regarding the compatibility of their sexuality with their faith. Aysha articulates this when she says that God didn't love her because she was gay and shares the admonishments she received from Christian friends. Her description of this internal conflict as ‘trying to square the circle’ highlights the perceived impossibility of reconciling these aspects of her self—a sentiment felt by others. Grace recounts her exclusion from the church community, whilst Asher describes his withdrawal from church due to judgement.

Although these participants faced rejection from their church communities, their personal faith revealed a distinction between communal religious practice and individual beliefs. Alistair's assertion that ‘God created him’ and Grace's reference to God as the source of love highlight a shift towards a more personal and less institutional understanding of faith.

Despite these struggles, statements such as ‘I carry them both along’, ‘I have a personal relationship with God and everything else is irrelevant’, and ‘I'm a gay Christian’ suggest a reconciliation of their sexuality and faith. For some, this reconciliation involved an exploration of theology outside of unsafe religious institutions. Others sought affirming communities within the church. This divergence emphasises the complex role of religion for LGBTQ+ people. It can be a protective factor, as seen with Aysha, or become a source of pain and isolation when it is non‐affirming.

**FIGURE 7:**
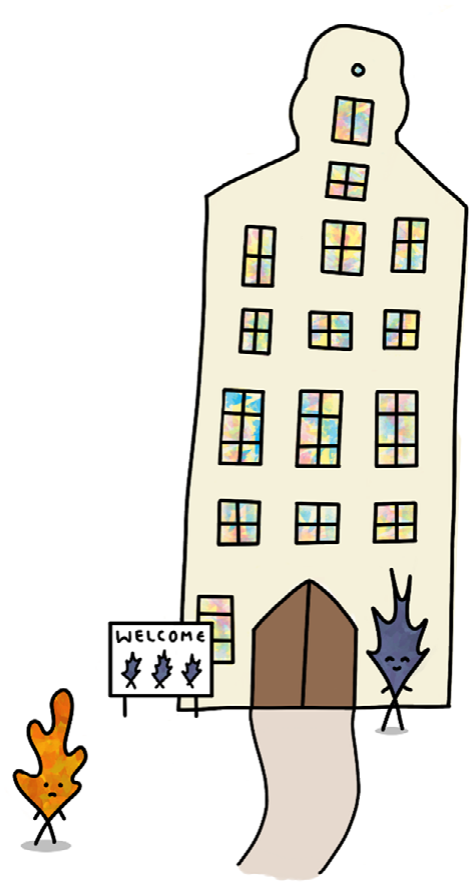
Rejection from church.

**FIGURE 8:**
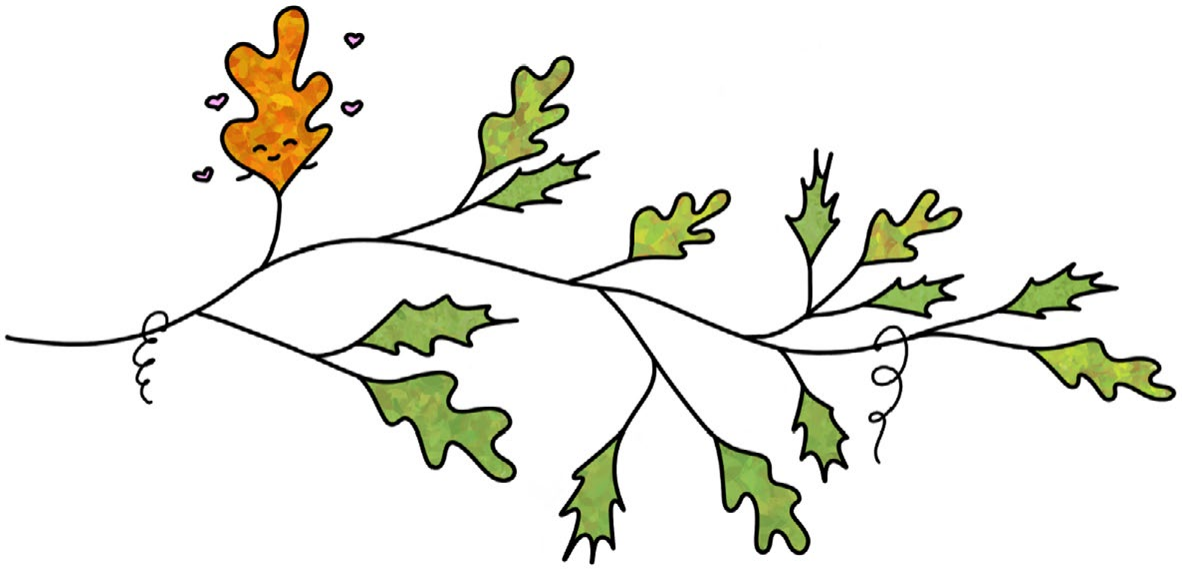
Reconciliation with God.

#### Feeling unworthy and unlovable

The impact and significance of this theme necessitate its treatment as a separate GET; however, it connects with others, such as ‘Feeling Safe in Relation to Others’, ‘Coming Out’, and ‘Is It Wrong to Be LGBTQ+?’

Participants reported that their experiences negatively impacted their self‐esteem, leading them to feel unworthy and unloved. Alex attributed these feelings to messages from society (‘it was the message from society was sort of, ‘you are gross’ and ‘God thinks you're an abomination’). Similarly, Liam's experiences of homophobic bullying led them to perceive themselves as ‘weird’. The labels ‘gross', ‘abomination’, and ‘weird’ foster shame, and participants described feeling that there is something wrong with them. Internalised shame seemed to prevent participants from feeling deserving of love, reinforcing a belief that they are inherently unlovable.

Ben's early relationships contributed to ‘self‐doubt and probably a lot of self‐loathing’. The rejection of his sexual identity by his parents led him to internalise their anger, resulting in self‐deprecation. He described himself as ‘some form of abomination’, reflecting that internalised shame made it difficult to love that part of his identity.

Theo's self‐esteem was affected by conversion therapy. He described feeling ‘really shitty like it invalidated my existence…it invalidated that part of me, who I was really scared and fragile about, I trusted her with that’ and ‘I thought I was broken for a long time.' The term ‘broken’ conveys his sense of feeling flawed and difficulties in self‐belief. He also suggested that the experience of conversion therapy contributed to his internalised homophobia and discriminatory attitudes towards other LGBTQ+ individuals, illustrating how these feelings of unworthiness were projected onto others.

Collectively, these experiences illustrate how internalised shame and negative self‐views impacted participants feelings of love and acceptance towards themselves.

**FIGURE 9:**
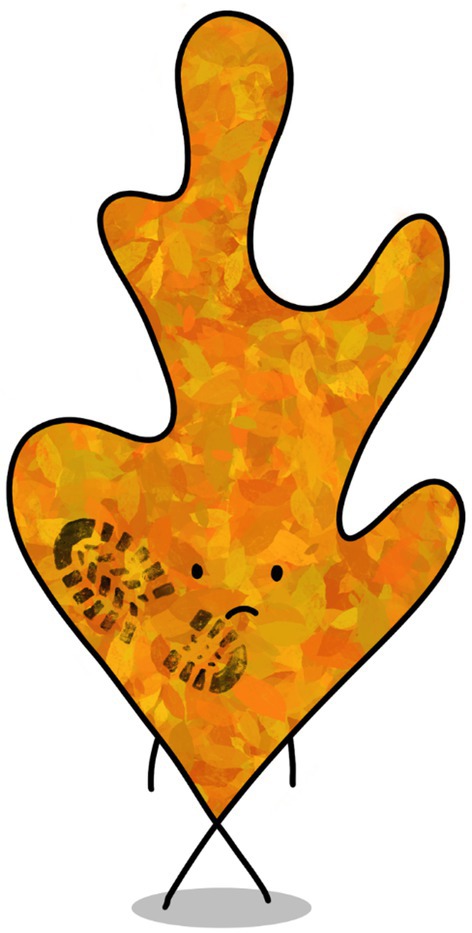
Feeling unworthy and unlovable.

#### Being authentically me

Participants described being on a journey and that along the way they had learnt the significance of being their authentic whole self. Three subthemes were identified that highlight how participants embraced being their authentic selves.

**FIGURE 10:**
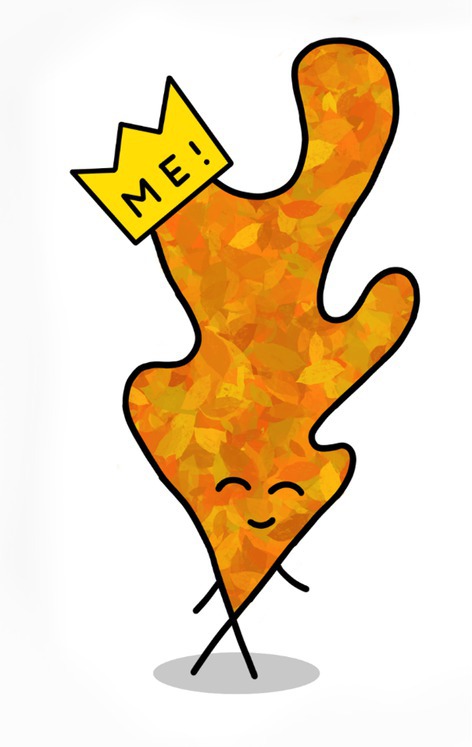
Being authentically me.

##### Feeling free versus fitting in

This subtheme encapsulates the divergence between participants as they battled between feeling free and fitting into a heteronormative society.

Oliver had a desire to fit in, but his focus was on belonging to a group. He says ‘I just tried to like fit into other people's identities’ which suggests that he felt it was better to have a social group than to stand out and risk rejection.

Ben tried to fit in with people's perceptions of his sexuality as he says ‘there was this huge dichotomy where I was at university and I was me. I was my true authentic self. And then I'd go home’. This encapsulates the struggle between a desire to fit in and belong in heteronormative society versus a desire to be the authentic self. This is a position that participants seem to oscillate between, but throughout the interview, participants shared how they moved towards feeling free.

No longer wanting to hide or pretend was a shared experience for participants, and it seemed that although fitting in may have some benefits, it felt like a burden. Participants talked about being ‘free’, which suggests that fitting in gave them a sense of imprisonment.

**FIGURE 11:**
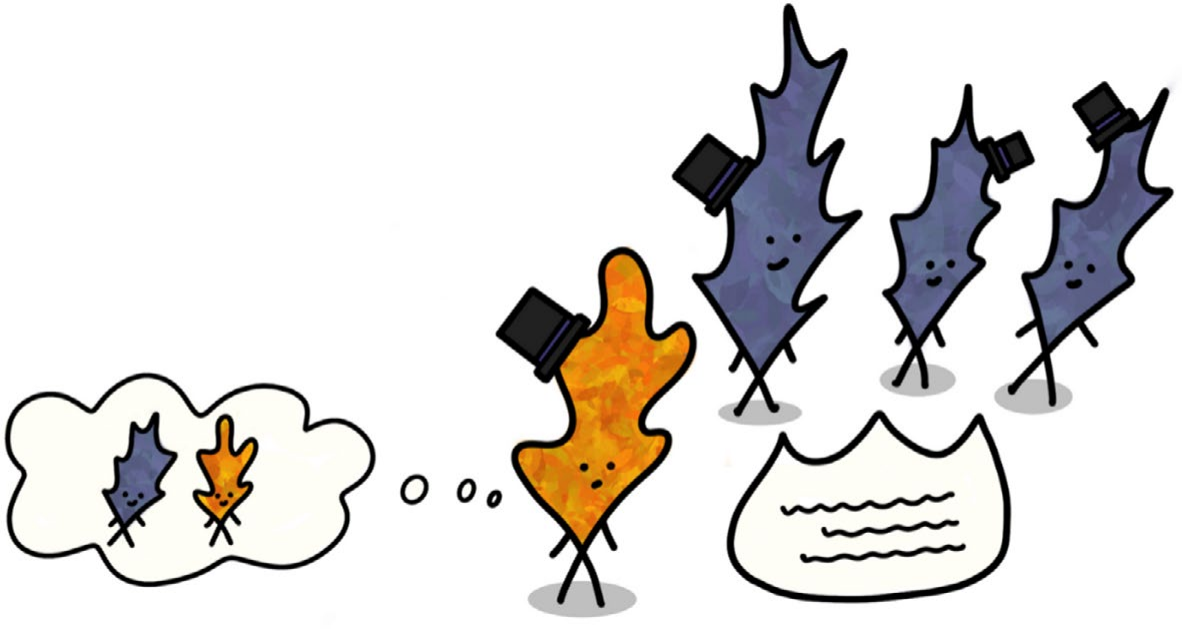
Fitting in.

**FIGURE 12:**
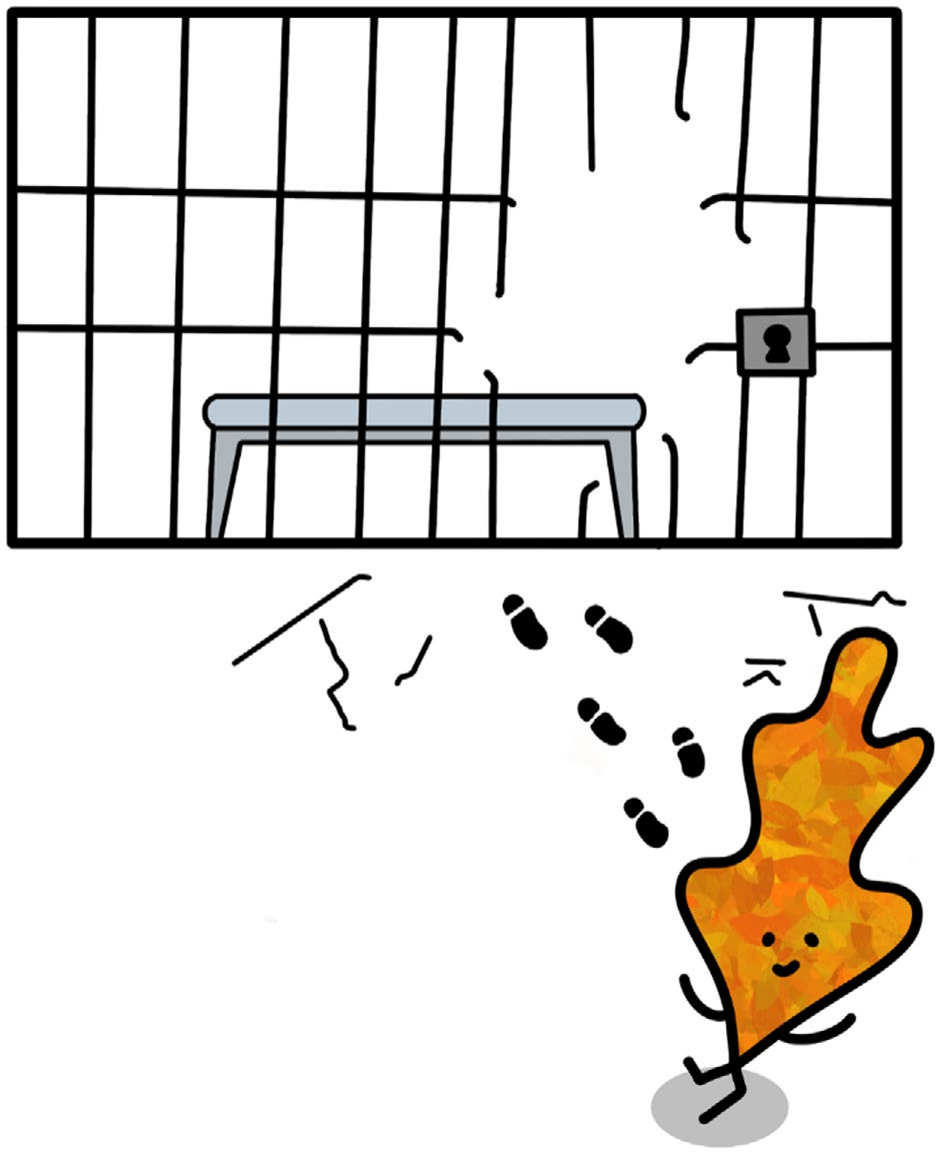
Feeling free.

##### Relearning relationships through experiencing safety and acceptance

In contrast to the subtheme of ‘Feeling Threatened’, participants shared experiences of relearning relational patterns and feeling safe and accepted as they experienced their sexual identity being affirmed and accepted.

Grace discovered that affirmative relationships within her church community contributed to her feelings of safety and acceptance.

Therapy played a significant role in acceptance for Liam and Theo, as they shared that it helped them accept their sexuality and navigate present relationships. The therapeutic relationship provided a trusting space, further enhancing learning.

Conversely, Jenny and Aysha found that independent self‐exploration was beneficial. Jenny metaphorically described her journey as ‘emerging from a cocoon…I've done this internal discovery’. This cocoon metaphor can be extended to others' experiences, highlighting the importance of retreating to safety and self‐exploration to disentangle past and present.

Overall, participants found that positive relational experiences, self‐exploration, and open conversations were instrumental in accepting their own sexual identity and sharing it with others in relationships. Overall, this helped participants feel safe and loved.

**FIGURE 13:**
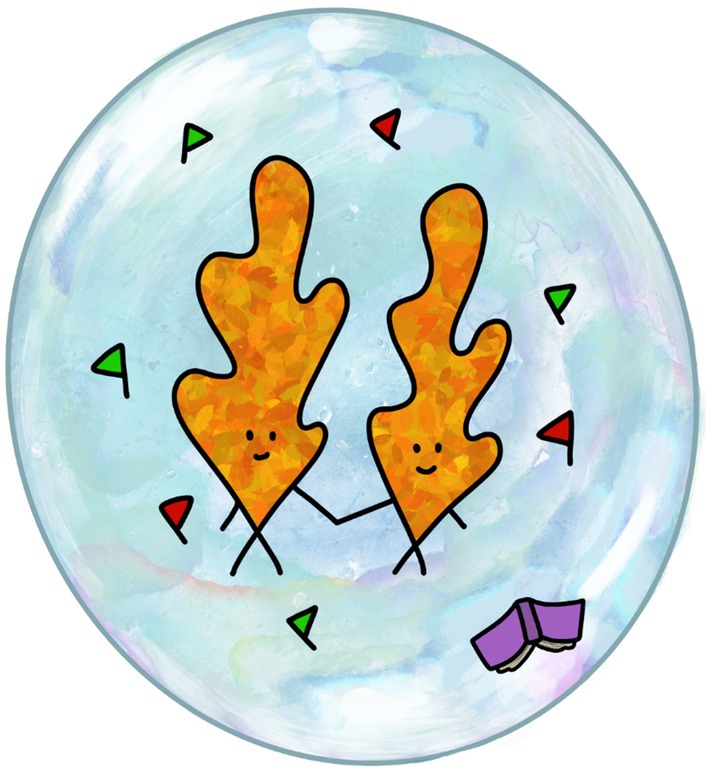
Relearning relationships through experiencing safety and acceptance.

##### Finding strength in myself versus finding strength in community

This subtheme highlights the differences in participants' experiences in finding the strength to be authentic, as some found strength through community whilst others relied on inner resilience.

Tracy states, ‘I was the strongest coping mechanism I had’, which emphasises her independent resilience and the significance of her inner strength. Similarly, Grace and Asher also valued independence and personal determination, recognising the importance of controlling their actions and trusting in their authenticity. Asher describes this as ‘standing strong’, evoking an image of steadfastness amid struggles. Notably, all these participants share a Christian faith; perhaps spiritual guidance and a sense of purpose contributed to their inner strength.

For other participants, finding strength in community increased their confidence to be authentic. Eight participants emphasised the positive impact of affirming friendships within the LGBTQ+ community. These relationships fostered camaraderie and shared experiences, as Liam articulates, ‘it was like, ‘oh yeah, me too’. Affirming communities provided encouragement and support, which participants described as liberating. Asher's experience of being ‘welcomed into the community’ evokes an image of open arms accepting him. This communal support emboldened participants to express themselves freely, such as wearing what they wanted and exploring hobbies. Finding community also counteracted previous negative experiences of being labelled ‘weird’ and ‘abnormal’, as participants saw they were not alone.

For Aysha, being part of an affirming LGBTQ+ church community gave her a sense of joy and freedom, as it allowed her to be authentic in all aspects of her life. Ben's account reflects the profound impact of community, as he shares, ‘I have met the best people in my life…they have stopped me from killing myself’, suggesting that a supportive community provided him with the strength to overcome loneliness.

**FIGURE 14:**
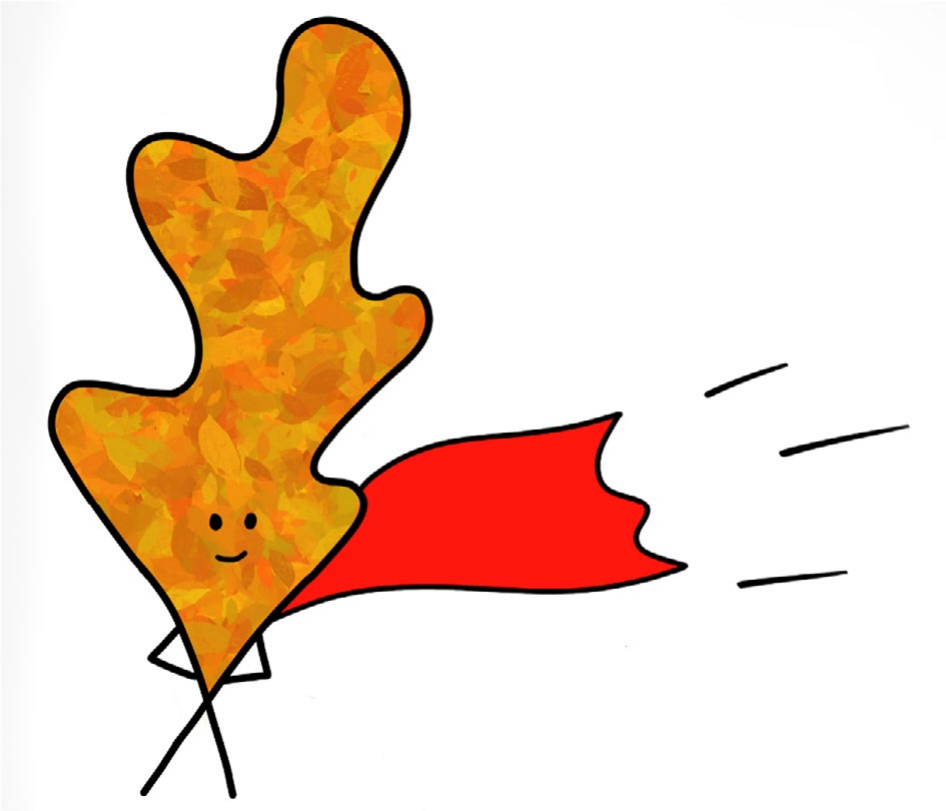
Finding strength in myself.

**FIGURE 15:**
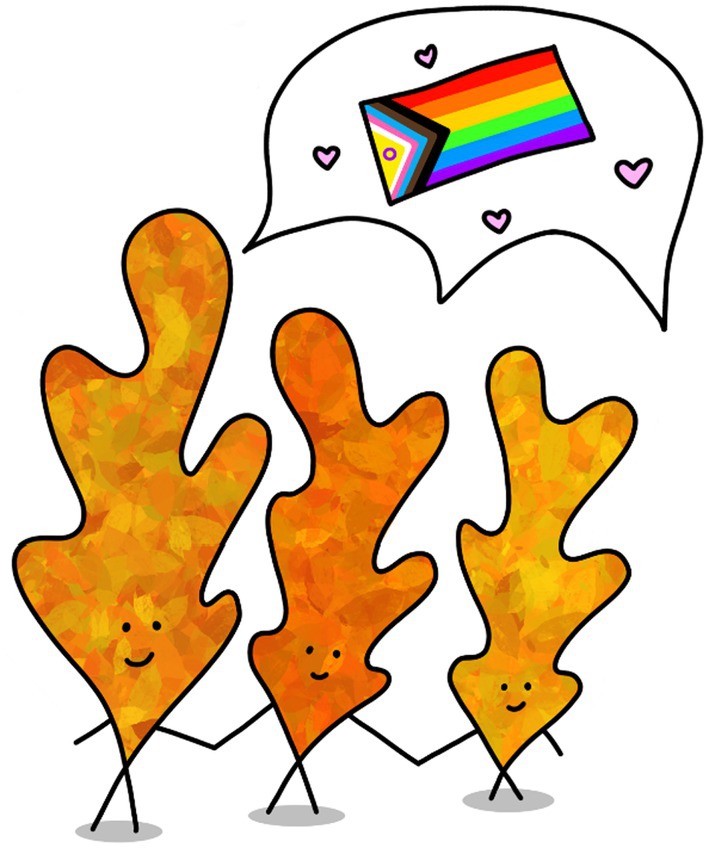
Finding strength in community.

## DISCUSSION

The aim of this study was to explore the relational experiences of LGBTQ+ individuals through a CAT lens. Six key themes were identified: *Feeling threatened*; *Coming Out*: *Discovering and Sharing Who I Am*; *Is it Wrong to be LGBTQ+? Questions and Beliefs about Being ‘Normal’*; *Different Parts of Me*; *Feeling Unworthy and Unlovable*; and *Being Authentically Me*. Subthemes were also identified within these.

The GETs highlight the impact of early caregiver relationships, coming out, and societal experiences on participants' self–self relationships. They also reveal the complexity of sexual identity, highlighting intersections with neurodiversity and religion. These findings align with existing literature, which emphasises the importance of caregivers' responses to sexuality (Taylor & Neppl, 2023) and demonstrates how negative societal reactions can impact relationships with the self and others through homophobia (Frost & Meyer, 2009). Participants described coming out as fluid and complex, supporting critiques of linear coming out models (D'Augelli, 1994; McLean, 2007), suggesting that moving beyond these may be beneficial.

Gordon and Silva (2015) highlighted that sexual identity involves making sense of cultural and social experiences, a concept reflected in these findings as participants described the impact of societal and cultural messages about religion, sexuality, and neurodiversity. The results highlighted the negative impact of rejection from religious communities, but also the significance of strengthening a personal relationship with a God. However, participants did not share experiences of the intersection of sexual identity and ethnicity. This contrasts with literature which suggests that LGBTQ+ individuals from ethnically minoritised backgrounds face increased psychological distress and identity concealment due to multiple identity‐related oppression (Salerno et al., 2023). Perhaps, the inherent power dynamic within the interview and the ethnicity of the interviewer (white British) impacted these conversations. Despite challenges, participants' experiences highlight resilience and the importance of self‐discovery and community in embracing their authentic self.

This research applied CAT theory as an additional framework to gain a deeper understanding of the GETs by exploring relational influences on self‐development and how this may be illustrated through RR and RRPs.

### Mapping the findings

Mapping in CAT facilitates the identification of RR and RRPs and enhances our relational understanding of the contexts in which we exist (Potter, 2020). The resulting map forms an ‘Orchestral Story’ (Potter, 2020), as it encapsulates multiple narratives from the GETs. Despite their individuality, these stories collectively contribute to a greater harmony, illustrating how mapping serves as a synthesis of experiences.

**FIGURE 16:**
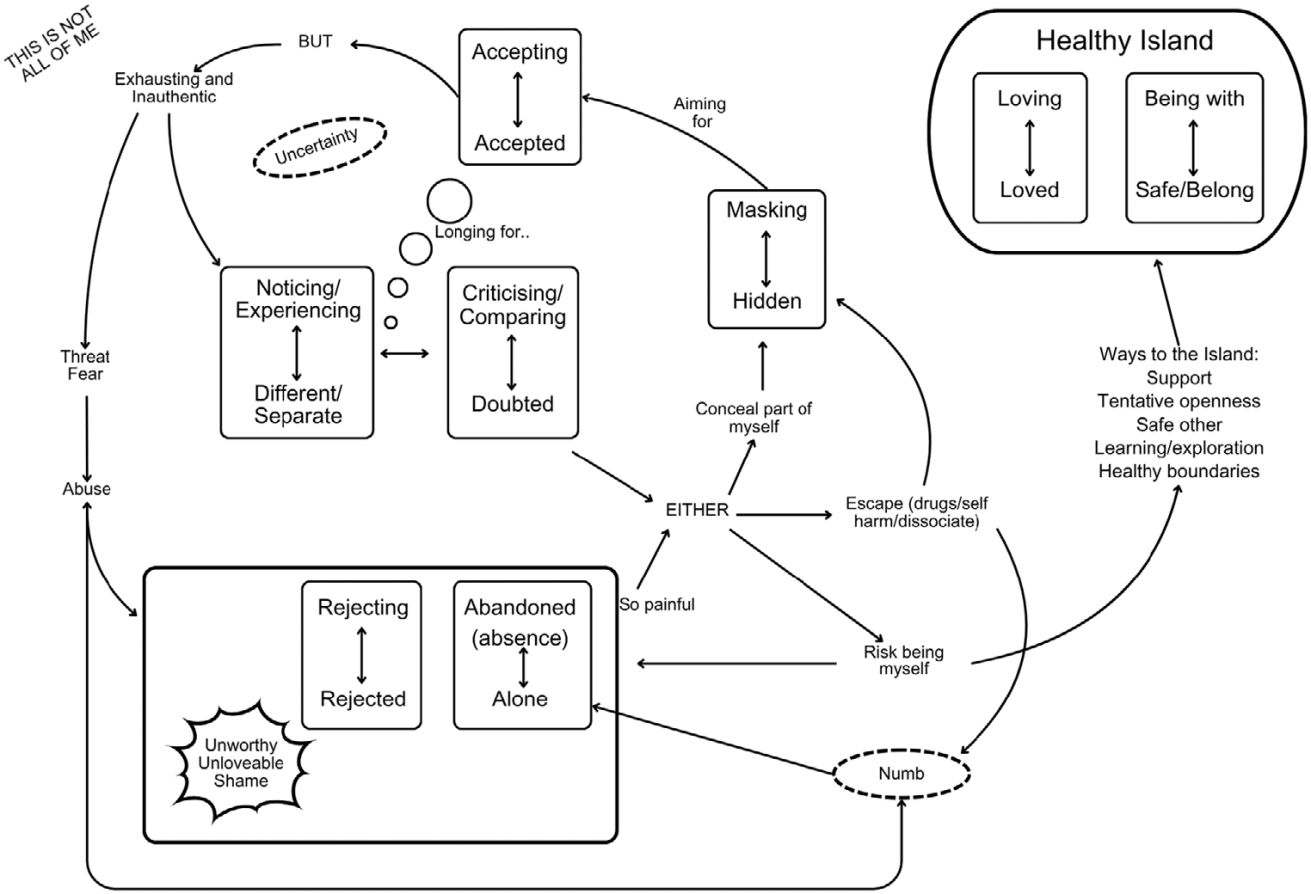
CAT map.

Within the map, each RR or RRP exists on a spectrum. For some participants, experiences of rejection stem from abuse, whilst for others, these feelings arose from ongoing microaggressions encountered in familiar settings, such as pubs or church. All these experiences are painful and contribute to the dynamics represented on the map, but the author wishes to remind the reader that these patterns and experiences do not look or feel the same for all LGBTQ+ people. See Figure 16 for the map created.

Whilst the map does not have a definitive ‘starting point’, the RRs of noticing and experiencing differences were common in early life. Participants became aware of difference through society and peers, which led to feeling separate. As participants experienced judgement and homophobic messaging from a heteronormative society, this awareness led to a critical and comparative RR, which facilitated self‐doubt. Ryle and Kerr (2020) emphasise that failure to address sexuality can restrict a child's development and induce feelings of guilt and doubt, as the noticing/experiencing is not affirmed. This dynamic played a significant role in the learning process and often resulted in internalised homophobia, as the RR criticizing/comparing‐doubted became an internalised self–self relationship, which was frequently accompanied by a longing for acceptance. Part of this desire for acceptance involved a longing to be heterosexual, neurotypical, or atheist—a fantasy that reflects the lived reality of others.

### RRPs and the identity solution

Potter (2020) emphasises that individuals may occupy a ‘bad but tolerable’ position on the map; a space where someone retreats for safety by adopting an identity solution that, whilst less painful than confronting their core pain, still involves sacrifices. For some participants, this conflict emerged from the perceived incompatibility between their sexuality and religious beliefs (Gordon & Silva, 2015), whilst for others, it stemmed from social interactions that implied their sexuality was unacceptable.

This RRP of self‐protection begins with a dilemma where an LGBTQ+ individual must either choose to risk being themselves or hide part of themselves through escapism or concealment. Concealing leads to masking the different or incompatible part of the self. The word ‘masking’ was chosen as this demonstrated the intersectionality of the concealing of faith, neurodiversity, gender, and sexuality that occurred. Additionally, this was often a conscious choice for participants as it allowed parts of themselves to be hidden and yet safe. One way Tinlin‐Dixon (2023) describes this RR is denying‐cut off, which resonates with the sacrifice participants made as they masked parts of themselves. This identity solution led participants to modify their appearance, choice of partners, speech, and other behaviours to feel accepted.

Participants coped with difficult emotions by escaping through drugs, alcohol, dissociation, and self‐harm. This allowed them to feel numb so they could be masked‐hidden or have the energy to maintain a masked identity. Whilst this RRP helped them survive by alleviating pain as they felt accepted, the initial refuge that this provided soon becomes a prison (Potter, 2020), as participants shared feelings of exhaustion, entrapment, and inauthenticity. Participants shared being hypervigilant amidst the uncertainty of the RRP, as they oscillated between noticing/experiencing difference and feelings of self‐doubt, exhaustion, and threat. For some, participants repeatedly experienced this dilemma as they grappled with the decision of whether to continue masking, seek escape, or risk expressing their authentic selves.

The inclusion of the phrase ‘this is not all of me’ serves as a reminder that the map offers only a partial view of the stories. As Potter (2020) suggests, this can reinforce that the map is a means of hovering and shimmering, meaning it can provide a space to pause and reflect on certain experiences, and acknowledge significant aspects of the narrative, without reducing identity to these hardships alone.

### Core pain

Core pain refers to the often unmanageable and deep feelings that result from painful RRs (Ryle & Kerr, 2020). Abuse, fear, and losing family and friends were a reality for participants, which created RRs of rejecting‐rejected and abandoned‐alone. These RRs contributed to core pain: feeling unworthy and unlovable and shame. Participants tried to survive their core pain by escaping or finding coping mechanisms. Whilst the initial experiences of rejection came from family, friends, and society, over time this rejection became internalised. The external voices of others transformed into an inner voice, leading participants to reject parts of themselves, such as their sexuality (Ryle, 1975; Ryle & Kerr, 2020).

### Exits and the healthy island

Wilde McCormick (2017) introduces the concept of the ‘healthy self’; a self that is integrated and accepted. The ‘healthy island’ is a space where the healthy self resides, enabling the development of healing RRs. Tinlin‐Dixon (2023) introduces the notion of the ‘Queer Healthy Island’, a space where LGBTQ+ individuals can safely express their authentic selves. This resonated with participants, many of whom described their authentic selves as involving the integration of various aspects of their identity, including religion and neurodiversity.

Benson and Discepolo Ahmadi (2024) suggest that shifting away from harmful RRs for LGBTQ+ individuals is important for well‐being, proposing that the RR of accepting‐accepted acts as an exit to the healthy island and authentic self. However, we argue that this RR should not be the goal. As one participant noted, ‘being okay with parts of me should just be the baseline’, suggesting that mere acceptance should not form the foundation of the healthy island. Instead, the healthy island creates a space where the loving‐loved RR can be formed, allowing participants to embrace and love their authentic selves.

The loving‐loved RR is self‐to‐self and other‐to‐self. For some, being loved within a community was crucial; for others, self‐love or love from God was key. This RR reflects Bowlby's (1988) concept of secure attachments, particularly ‘earned secure attachment’ which emphasises that experiencing love and safety from others in adulthood can help repair insecure relational patterns and psychological well‐being (Saunders et al., 2011). The opportunity to form secure attachments later in life and feel safe in relationships seemed pivotal in participants' journeys towards the healthy island.

Ryle (1975) states that the self is shaped through interactions with significant others; therefore, as differences are expressed and valued within these RRs, the RR is internalised into a loving self‐to‐self relationship. This helped participants to heal from their core pain and feel free to love their more connected and authentic self as others did. Overall, the presence of a compassionate safe other provided an exit to the Healthy Island and enabled participants to relearn how to love others and their authentic selves.

### The socio‐political context

CAT theory posits that the self is a psycho‐bio‐social identity shaped by cultural contexts (Ryle & Kerr, 2020). Consequently, awareness of socio‐political and historical contexts was central to the map, as participants experienced homophobia, internalised homophobia, transphobia, and prejudice. For example, participants were exposed to non‐affirming religious ideologies, homophobic messages in the media and television, and political discourse that challenged or undermined LGBTQ+ rights. As individuals hover in and out of the map, they can encounter the voices of their external social and political realities, which shaped their experiences (Ryle & Kerr, 2020).

Recent research highlights that CAT provides a framework for incorporating the broader social context into therapy, and recognising and validating these factors is a critical starting point (Brown, 2024; Laws, 2019). This map demonstrates how society can function as the ‘other’, forming society‐self relationships, thereby integrating social context directly into the map and the RRs. This approach creates a more affirmative meaning‐making process for the LGBTQ+ community by acknowledging experiences of discrimination and offering a sense of empowerment through a shift from ‘being done to’ towards ‘doing with’.

### Clinical and research implication

The integration of the CAT framework and creation of a CAT map aims to help clinicians in supporting LGBTQ+ individuals experiencing mental health challenges related to their relational experiences. The CAT map offers an illustration of how RR and RRPs may be explored to address these challenges meaningfully and highlight possible exits to the healthy island. The collective reformulation letter was drafted to capture participants' shared narratives and was shared with them for feedback. This letter can also serve as a training tool for reformulation in CAT for LGBTQ+ individuals.

The findings open the door for future research to explore the application of the CAT map within a clinical context, with next steps involving case studies and single‐case experimental design. Such exploration could contribute to the development of a specialised CAT framework, tailored to the needs of the LGBTQ+ community.

### Strengths and limitations

Collaboration with the LGBTQ+ community emphasised inclusion and equality within a group that often experiences discrimination. This involvement contributed to the hermeneutic circle and enhanced the quality of the research by ensuring it remained focused on issues that were important to participants.

Commitment to diversity was demonstrated using a purposive sampling strategy, enabling a representation across a range of sexualities, genders, ethnicities, ages, classes, and religions. Despite this diversity, the author acknowledges that further progress is needed to ensure people from different backgrounds, particularly those from various religious and ethnic groups, feel safe sharing their experiences.

Furthermore, the age range of the sample was limited to 20–54. These people will have grown up in specific contexts that do not reflect the experiences of those before or after them. Specifically, some of the sample will not have lived through Section 28, and it is important to acknowledge that specific cohorts within minoritised communities will have experienced different trauma. Future research could explore how experiences vary across cohorts and the meanings individuals have made of them, in particular thinking about differences across RRs and RRPs.

This study was conducted in accordance with Yardley's (2000) principles, which ensured that the research remained meaningful and credible throughout. By maintaining a commitment to methodological rigor, this study has created a foundation for future research that may yield broader implications.

An important component of this research involved inclusive and collaborative dissemination (NIHR, 2021). To enhance accessibility—regardless of educational background, social class, language proficiency, or neurodivergence—a storyboard of illustrations capturing key themes in the data has been created and shared widely.

## CONCLUSION

This research identifies key themes pertinent to LGBTQ+ people, their emotional states and relational patterns; ultimately resulting in an ‘orchestral story’ within the CAT map. The map captures some of these distinct RR and RRPs and emphasises the role of the society‐self relationship in identity formation. Moreover, it highlights the importance of the ‘healthy island’ and illustrates how healing RRs create opportunities for individuals to embrace and love a more connected self.

## AUTHOR CONTRIBUTIONS

**Deborah Charis Bell:** Investigation; writing – original draft; methodology; formal analysis; project administration; software; data curation; writing – review and editing. **Rowan Tinlin‐Dixon:** Conceptualization; writing – review and editing; supervision; methodology.

## CONFLICT OF INTEREST STATEMENT

None.

## DISCLOSURE

The images used within this thesis are a paid‐for commission with © 2025 Matilda Cowen. All rights reserved. Images are used under strict permission from the artist for this thesis only. Any unauthorised usage, adoption or adaption is strictly forbidden. If you would like to use the images for your clinical work, please contact the corresponding author. For enquiries with the artist, you can contact the corresponding author.

## Supporting information


Figure S1.

Figure S2.

Figure S3.

## Data Availability

The data that support the findings of this study are available on request from the corresponding author. The data are not publicly available due to privacy or ethical restrictions.
