# Supplementary figures and images for: Exploring relational and emotional experiences of the LGBTQ+ community through a cognitive analytic therapy lens
Source: Psychol Psychother. 2025 Nov 29;99(2):363–85. doi: 10.1111/papt.70016 (PMC13162184; doi:10.1111/papt.70016)

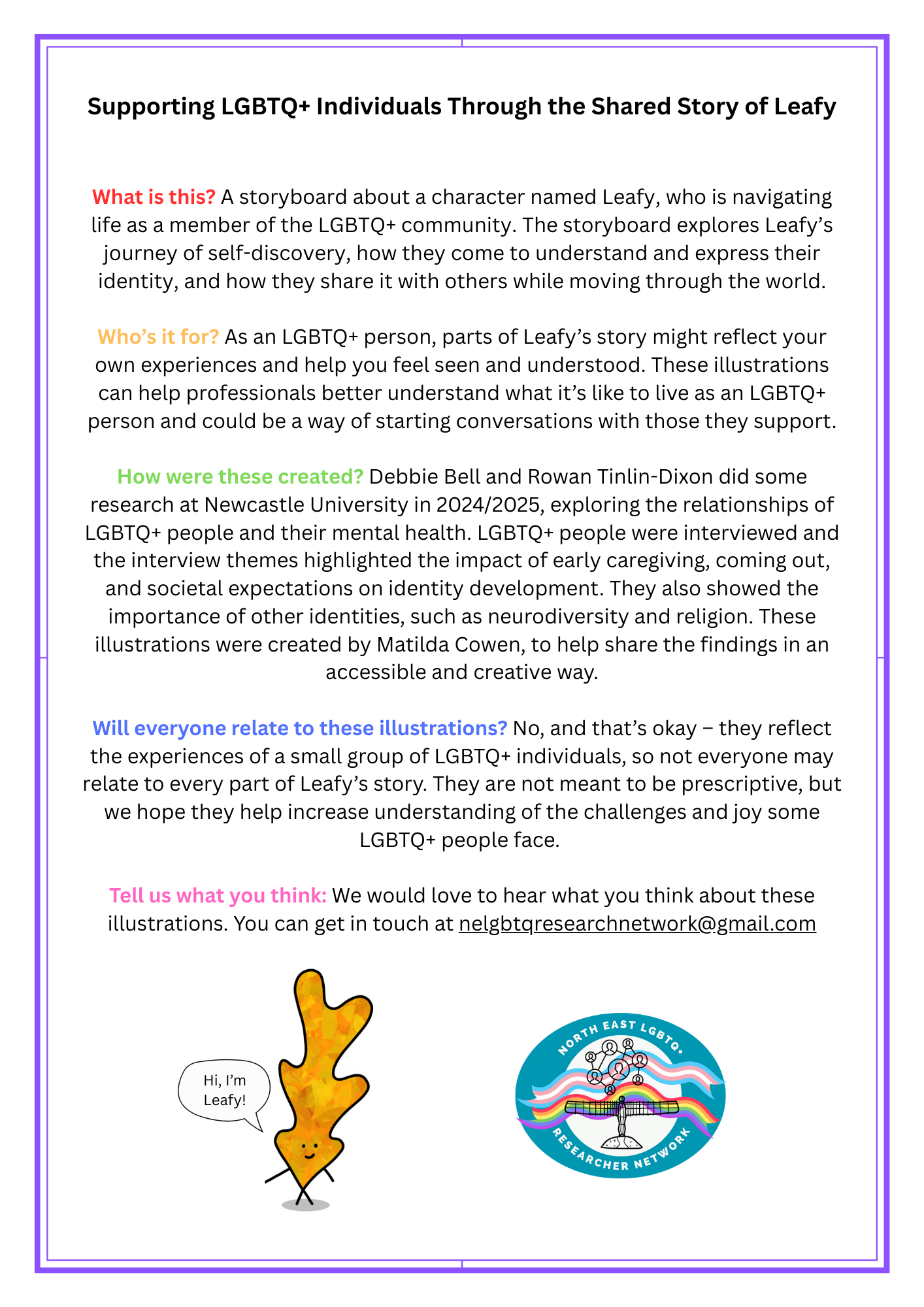

Supplement: Supplementary file 1 — Figure S1. [file PAPT-99-363-s001.png]

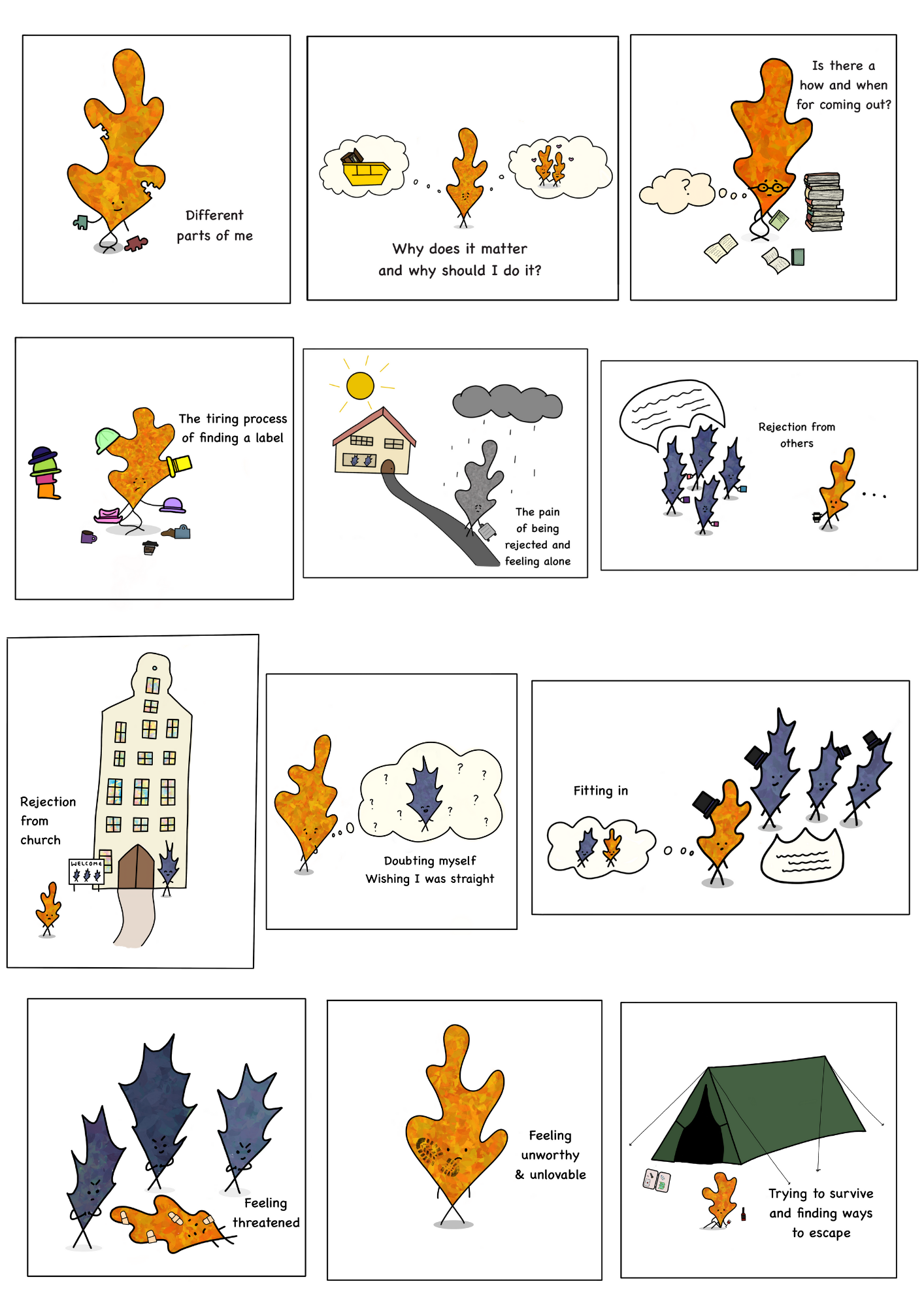

Supplement: Supplementary file 2 — Figure S2. [file PAPT-99-363-s002.png]

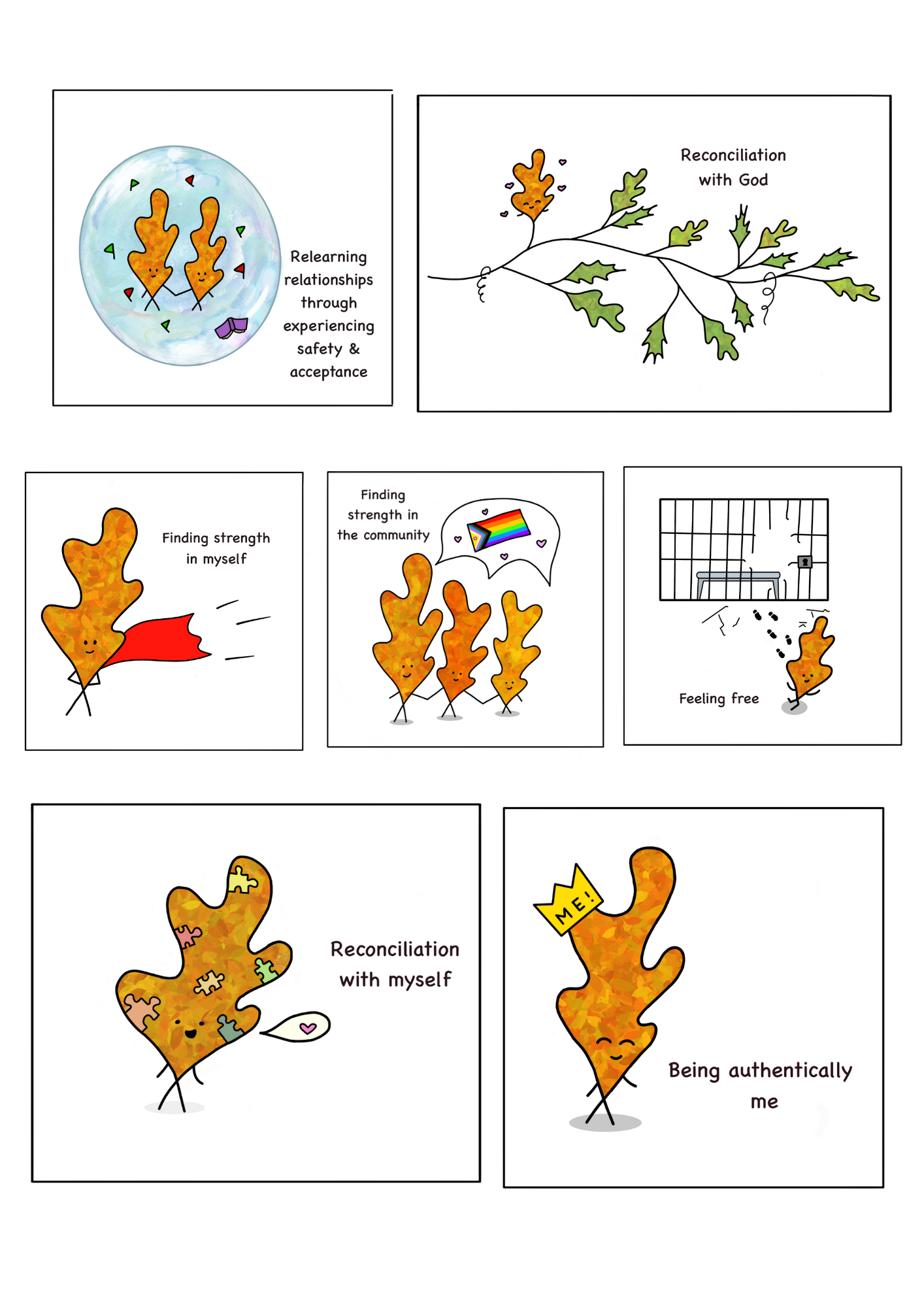

Supplement: Supplementary file 3 — Figure S3. [file PAPT-99-363-s003.png]
